# Segmentation of MR image using local and global region based geodesic model

**DOI:** 10.1186/1475-925X-14-8

**Published:** 2015-02-19

**Authors:** Xiuming Li, Dongsheng Jiang, Yonghong Shi, Wensheng Li

**Affiliations:** Digital Medical Research Center, School of Basic Medical Sciences, Fudan University, Shanghai, 200032 PR China; Department of Anatomy, Histology and Embryology, School of Basic Medical Sciences, Fudan University, Shanghai, 200032 PR China; Shanghai Key Laboratory of Medical Imaging Computing and Computer-Assisted Intervention, Shanghai, 200032 PR China

**Keywords:** Intensity inhomogeneity, Level set method, Local image information, Global image information

## Abstract

**Background:**

Segmentation of the magnetic resonance (MR) images is fundamentally important in medical image analysis. Intensity inhomogeneity due to the unknown noise and weak boundary makes it a difficult problem.

**Method:**

The paper presents a novel level set geodesic model which integrates the local and the global intensity information in the signed pressure force (SPF) function to suppress the intensity inhomogeneity and implement the segmentation. First, a new local and global region based SPF function is proposed to extract the local and global image information in order to ensure a flexible initialization of the object contours. Second, the global SPF is adaptively balanced by the weight calculated by using the local image contrast. Third, two-phase level set formulation is extended to a multi-phase formulation to successfully segment brain MR images.

**Results:**

Experimental results on the synthetic images and MR images demonstrate that the proposed method is very robust and efficient. Compared with the related methods, our method is much more computationally efficient and much less sensitive to the initial contour. Furthermore, the validation on 18 T1-weighted brain MR images (International Brain Segmentation Repository) shows that our method can produce very promising results.

**Conclusions:**

A novel segmentation model by incorporating the local and global information into the original GAC model is proposed. The proposed model is suitable for the segmentation of the inhomogeneous MR images and allows flexible initialization.

## Background

Magnetic resonance image can provide excellent spatial resolution and superb soft tissue contrast for anatomical and functional structures. Accurate segmentation of MR image is an essential step in medical image analysis. However, the intensity inhomogeneity due to the unknown noise and weak boundary makes the segmentation a challenge. Various segmentation algorithms have been proposed in the literature. Particularly, Active contour model (ACM) receives the widespread attention since it can provide promisingly smooth and closed contours to cover object boundaries with sub-pixel accuracy [1, 2]. The existing ACMs can be mainly categorized into two classes: edge-based models and region-based models.

One of the most popular edge-based models is geodesic active contour (GAC) model [3, 4], which utilizes image gradient to construct an edge stopping function (ESF) to keep the contour evolution within the object boundaries. The model has been successfully applied in the general images with strong object boundaries, but it may suffer from boundary leakage in the brain MR images which typically contain weak boundaries due to low contrast and partial volume effect. However, when the initial contour is far away from the desired object boundary, the GAC model will fail to find the target [5]. And then Song proposed an edge-based ACM that is driven by the regularized gradient flux flows [6]. The method is not only robust to noise, but also preserves the edge information, thereby achieves accurate segmentation results.

Region-based models have many advantages over the edge-based ones. For example, region-based models are less sensitive to noise and contour initialization since they utilize region information as a substitute for image gradient to constrain contour evolution. Moreover, they can successfully segment images with the weak boundaries or even without boundaries. The well-known region-based model, Chan-Vese (CV) model, assumes that image intensities are statistically homogeneous in each region, and therefore it fails to segment MR images with intensity inhomogeneity [7]. Then Li et al. proposed a local binary fitting (LBF) model to overcome the intensity inhomogeneity. The LBF model can provide desirable segmentation results because it uses the mean of the local region information. However, it is sensitive to the initial contours and is easy to trap into a local minimum, which limits their practical applications [8, 9]. Zhang et al. proposed a maximum likelihood in transformed domain method to simultaneously segment images and correct bias field. The method demonstrated the superiority by taking the mean and variance in a local region into account [10].

For making full use of the advantage of the three methods mentioned above, some hybrid models combining the local and global intensity fitting energies were proposed to drive the evolution of initial contours. Lei He et al. blended GMM model, Hueckel model and CV model, and finished the segmentation of inhomogeneous image, but is prone to be sensitive to initialization and parameterization [11]. Zhang et al. proposed an improved method of GAC model, named as GCV model, which utilizes the global intensity information to construct a signed pressure force (SPF) function to drive the contour evolution. This method also proposed a new level set function re-initialization method, i.e., selective binary and Gaussian filtering regularized Level Set, which is robust and simple to implement, but is hard to deal with the image having inhomogeneous gray intensity or weak boundary images [5]. Wang et al. proposed a hybrid level set method which has a LBF term based on the local intensity fitting and CV term based on an auxiliary global intensity fitting. Due to combining the local and global intensity information, the proposed model can avoid trapping into a local minimum [12].

Motivated by Zhang et al. and Wang et al. method, the paper proposes a novel method based on the GAC model. Concretely, a new SPF function is defined as an adaptive combination of the local and the global fitting terms. Local term is the local part being responsible for attracting the contour toward the object boundaries, and the global one is the auxiliary global part incorporating the global image information to drive the motion of the contour far away from object boundaries. Then the balance between the local and global fitting terms is dynamically adjusted by the weight calculated on the local intensity contrast. The proposed SPF function is regularized by a binary level set function to avoid the traditional re-initialization of the level set function to a signed distance function [5]. The proposed model is first presented as a two-phase level set formulation and then extended to a multi-phase formulation. After minimizing the energy function [13], the method is able to segment MR images. Experiments on synthetic and MR images show that the proposed method can not only overcome inhomogeneous gray intensity, but also deal with the images having complex background and weak boundary. Moreover, it reduces function’s dependence on initial contour.

The rest of the paper is organized as follows. We briefly review several classic models and their limitation in “Background”. The proposed method is introduced in “Methods”. We discussed our proposed method and compared our segmentation results with those of GCV method, LBF method and Li et al. method [14] in “Results and discussion”. Finally, some conclusive remarks are included in “conclusion”

### The related methods

Given the image *I*(*x*), *x*∈Ω and *Ω* ⊂ *R*^2^. Let the initial contour *C*(*q*):[01] → *R*^2^ be a parameterized planar cure in Ω. In this subsection, we will introduce GAC model, CV model, GCV model and LBF, respectively.

### The GAC model

The traditional edge-based GAC model [3, 4] is formulated as,
1

where  is a positive, decreasing and regular edge stopping function (ESF) described as,
2

where ∇*K*_σ_*I denotes convolving image *I* with a Gaussian kernel, *K*_*σ*_, whose standard deviation is σ. σ is the scale parameter that controls the region-scalability from the locally small neighborhood to the whole image domain and is adaptively chosen in the images, similar to [9].

The GAC model utilizes image gradient to construct an edge stopping function (ESF) to stop the contour evolution on the object boundaries. For images of weak boundaries or the initial contour is far away from the desired object boundary, the GAC model will fail to find the target [5].

### The CV model

The region-based CV model [7] considered a special case where the original image intensity is assumed as homogeneous. The energy function of the segmenting curve *C* of CV model is defined as,
3

where *C*_1_ and *C*_2_ are two constants which are the average intensities inside and outside the curve *C*, respectively. λ_1_ and λ_2_ are nonnegative constants and control the driven force of the image data inside and outside the contour, respectively. By minimizing Eq. (3) *C*_1_ and *C*_2_ are solved as follows:
45

where *ϕ* (*x*) is the level set function and *H*(*ϕ* (*x*)) is the Heaviside function which is regularized as follows:
6

where *ϵ* is nonnegative constant. Similar to [7, 8], we set *ϵ =* 1.0 for good approximation of *H* by *H*_ϵ_.

**Figure 1:**
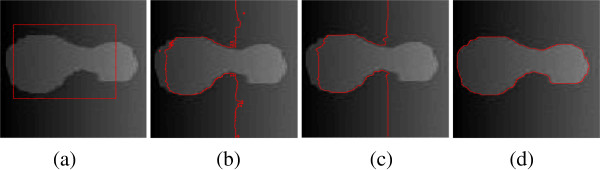
**Illustration of the experimental results on the synthetic image with intensity inhomogeneity. (a)** Original image and initial contour. **(b)** Result of CV model. **(c)** Result of GCV model **(d)** result of LBF model.

Obviously, *C*_1_ and *C*_2_ are related to the global properties of the image contents inside and outside the contour, respectively. Without taking local image information into account, the CV model fails to segment images with inhomogeneity. Figure 1(a) shows a synthetic image with intensity inhomogeneity. The CV model fails to segment this image, as show in Figure 1(b).

### The GCV model

**Figure 2:**
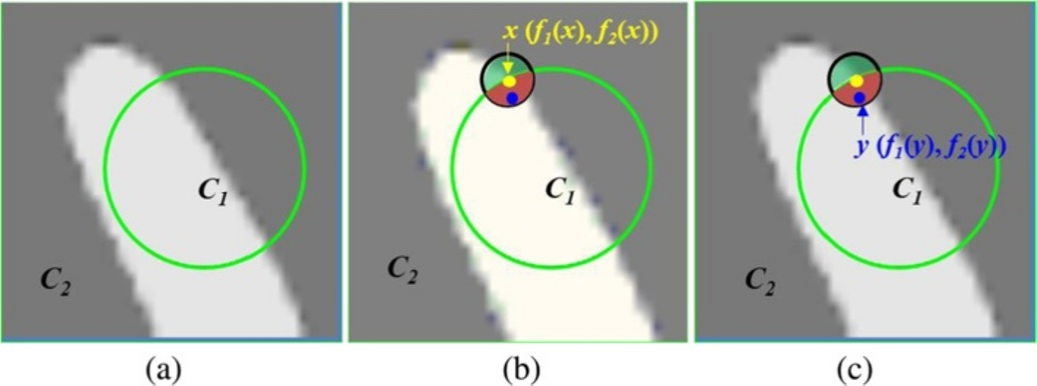
**Graphical representation of one level set model.** The green line denotes the contour curve, which divides the image into two regions, interior region *C*_1_ and the exterior region *C*_2_ **(a)**. The local neighborhood of *x*, *K*_*σ*_(*y* - *x*) is represented by the black circle. The circle is spilt by the green curve into local interior (red) and local exterior (green) regions. The small yellow and blue dots represent the point *x* along the contour and point *y* in the local region of point *x*, respectively. *f*_1_(*x*) and *f*_2_(*x*) are computed in the local interior and local exterior region of the point *x* to fit the image intensities near the point *x* **(b)**. *f*_*1*_(*y*) and *f*_*2*_(*y*) are computed in the local interior and local exterior region of the point *x* to fit the image intensities near the point *y* **(c)**.

The GCV model [5] utilized the statistics of local region in CV model and proposed a signed pressure force (SPF) function [15] whose value is in the range of [-1, 1]. And GCV can modulate the signs of the pressure forces inside and outside the region of interest so that the contour shrinks when outside the object, or expands when inside the object. The SPF function is defined as follows,
7

where *C*_1_ and *C*_2_ are defined in Eq. (4) and Eq. (5), respectively. Substituting the ESF in Eq. (1) for the SPF function in Eq. (7), the improved region-based segmentation method was formulated as GCV. Figure 2(a) illustrates the interaction of the interior and exterior regions of the contour. The contour curve (green line) divides the image into the interior and the exterior regions of the contour, which are corresponding to *C*_*1*_ and *C*_*2*_ computed in Eq. (4) and Eq. (5)_,_ respectively.

The GCV model shares the advantages of the CV and GAC models. This model utilizes the statistical information inside and outside the contour to construct a region-based SPF function to substitute ESF. Under the effect of SPF function, the contour can shrink when it is outside the object or expand when inside the object. Moreover, the level set function of the GCV model is regularized by the selective binary and Gaussian filtering [5], and the computational complexity is decreased by comparing with the traditional level set methods. However, this method can’t deal with the inhomogeneous or fuzzy boundary images. For example, Figure 1(c) shows that the GCV model fails to segment the object correctly.

### The LBF model

The LBF model [8, 9] utilizes two spatially varying fitting functions *f*_1_(x) and *f*_2_(x) to approximate the local intensities on the two sides of the contour. And the image fitting energy function was defined as follows:
8

Due to the localization property of the kernel function, the contribution of the intensity *I*(*y*) to the LBF energy decreases to zero as the point *y* moves away from the center point *x*. Therefore, the LBF energy is dominated by the intensity *I*(*y*) of points *y* in the neighborhood of *x*. This localization property plays a key role in segmenting the images with intensity inhomogeneity. Figure 2(b) illustrates the interaction of the local interior and local exterior regions of the point *x*. The local neighborhood of *x*, *K*_*σ*_(*y* - *x*), is represented by the black circle. The circle is spilt by the green curve into local interior (red) and local exterior (green) regions. The small yellow and blue dots represent the point *x* along the contour and point *y* in the local region of point *x*, respectively. *f*_1_(*x*) and *f*_2_(*x*) are computed in the local interior and local exterior region of the point *x* to fit the image intensities near the point *x*.

By minimizing Eq. (8), *f*_1_(*x*) and *f*_2_(*x*) are solved as follows,
9

10Because of using local region information, specifically local intensity mean, the LBF model is able to provide desirable segmentation results even in the presence of intensity inhomogeneity. Figure 1(d) shows the correct result of the LBF model. The disadvantage of LBF model is that it is easy to fall into a local minimum, and then it is sensitive to the initial location of contour.

## Methods

### The design of novel SPF function

Our method implements a novel model which combines the advantages of GCV model and LBF model in a new SPF function by simultaneously taking the global and the local intensity information into account. Figure 2(c) shows the main idea of our method. Two fitting functions, *f*_1_(*y*) and *f*_2_(*y*), are computed in the local interior and local exterior region of the point *x* to fit the image intensities near the point *y*. In this way, for each center point *x*, the local SPF function can be minimized when the contour *C* is exactly on the object boundary. Here, our local SPF function is formulated as,
11

And the global SPF function is equalized to *S*(*I*(*x*)) in Eq. (7),
12

Then, our full energy function is formulated as,
13

where *ω* balances the local and the global fitting energies. Theoretically, *ω* should be set a larger value to make the weight of global SPF function bigger in the regions where intensity varies greatly, while set a smaller one in the regions where intensity varies smoothly. Following the work in [16], we adopt the adaptive weight,
14

where *β* is a fixed positive parameter and  is the average value of *C*_*R*_ over the whole image. *C*_*R*_ is a local contrast ratio of an image defined as,


where *R* defines the local window of size 5 × 5 centered at x. *ω* varies between 0 and 1 and reflects how rapidly the intensity changes in a local region. It is smaller in the smooth regions and bigger in the regions close to the boundary of objects. Therefore, the *ω* can adaptively adjust the global term in all regions.

Eq. (13) indicates that *S*^*New*^(*I*(*x*)) is in the range of [-1, 1] and can control the contour shrinks when it is outside the object, or expands when inside the object. Accordingly, it satisfies the requirement of being a symbols pressure function. Substituting the ESF in Eq. (1) for the SPF function in Eq. (13), the level set formulation of the proposed model is regularized as following [3]:
15

where *α* is the balloon force, which controls the contour shrinking or expanding.

### Extension to multi-phase level set model

**Figure 3:**
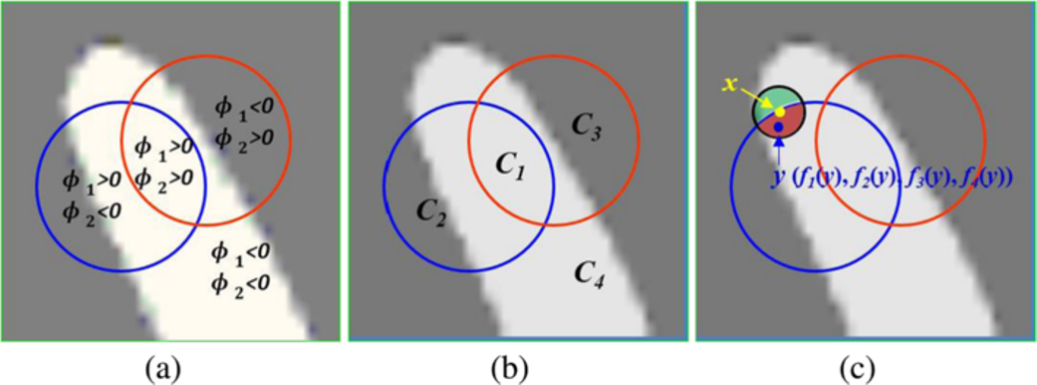
**Graphical representation of two level set model. (a)** Blue and red line are considered two zero level set function *ϕ*_1_ and *ϕ*_2_, which divides the image into four regions: *C*_1_, *C*_2_, *C*_3_ and *C*_4_ in **(b). (c)** The neighborhood of point *x*, *K*_*σ*_(*y* - *x*), is represented by the small black circle. The circle is spilt by the two level set function into local interior and local exterior regions. The small blue dot represents the point *y* in the local region of *x. f*_1_(*y*), *f*_2_(*y*), *f*_3_(*y*), *f*_4_(*y*) are computed in the local interior and local exterior region to fit the image intensities near the point *y*, respectively.

The model proposed above is a two-phase level set formulation, which is not able to segment multiple regions that are adjacent to each other. For example, in brain MR images, the regions of white matter (WM), gray matter (GM), and cerebrospinal fluid (CSF) may be adjacent to each other. An important application of this multi-phase formulation is for the segmentation of WM, GM and CSF. Therefore, we extend our model to a multi-phase level set formulation to segment multiple junctions. In multi-phase level set formulation, *n* level set functions can represent 2^n^ regions [17]. The multi-layer level set formulation can also represent multiple regions [18]. In the study, we focus on four-phase formulation, which is sufficiently to segment brain MR images. Two level set functions, ∅_1_ and ∅_2_, are used to define the partition of image domain as four disjoined regions [17]: {*ϕ*_1_ > 0, *ϕ*_2_ > 0}, {*ϕ*_1_ > 0, *ϕ*_2_ < 0}, {*ϕ*_1_ < 0, *ϕ*_2_ > 0}, {*ϕ*_1_ < 0, *ϕ*_2_ < 0}. Let *M*_1_ = *H*(*ϕ*_1_)*H*(*ϕ*_2_), *M*_2_ = *H*(*ϕ*_1_)(1 - *H*(*ϕ*_2_)), *M*_3_ = (1 - *H*(*ϕ*_1_))*H*(*ϕ*_2_), *M*_4_ = (1 - *H*(*ϕ*_1_))(1 - *H*(*ϕ*_2_)). Figure 3 shows the main idea of our method. The blue and red line denote the two zero level set function *ϕ*_1_ and *ϕ*_2_, which divides the image into four regions: *C*_1_, *C*_2_, *C*_3_ and *C*_4_ in Figure 3(b). The neighborhood of point *x*, *K*_*σ*_(*y* - *x*), is represented by the small black circle and it is spilt by the two level set function into local interior and local exterior regions. The small blue dot represents the point *y* in the local region of *x. f*_1_(*y*), *f*_2_(*y*), *f*_3_(*y*), *f*_4_(*y*) are computed in the local interior and local exterior regions to fit the image intensities near the point *y* according to the four regions, respectively.

Similar to GAC model, energy function is defined as,
16

where the local SPF function, , is defined as,
17

and the global SPF function, , is defined as,
18

and  can be similarly derived from Wang et al. [14] paper and Vest et al. [7] paper.
19

Minimizing the energy functional *E* in Eq. (16) with respect to *ϕ*_1_, we derive the gradient descent flow:
20

Likewise, minimizing the energy functional *E* with respect to *ϕ*_*2*_, we derive the gradient descent flow:
21

where *δ*(*ϕ*) is the Dirac function which is regularized as follows:
22

where *ϵ* is nonnegative constant. if *ϵ* is too small, the values of *δ*_*ϵ*_(*z*) tend to be near zero to make its effective range small, so the energy functional has a tendency to fall into a local minimum. The object may fail to be extracted if the initial contour starts far from it. However, if *ϵ* is large, although *δ*_*ϵ*_(*z*) tends to obtain a global minimum, the finial contour location may not be accurate. In “Results and discussion”, we will give some examples to show this drawback. We set *ϵ* = 0.3 for good approximation of *δ* by *δ*_3_.

### Implementation

We take two-phase segmentation as an example to describe the implementation. In traditional level set methods, the curvature-based term *div*(∇*ϕ*/(|∇*ϕ*|))|∇*ϕ*| is usually used to regularize the level set function and drive the contour evolution. Instead, Zhang et al. [5] utilizes a Gaussian filter to smooth the level set function for keeping the interface and getting rid of the curvature term. In addition, the term, ∇*s*^*New*^(*I*(*x*)) ⋅ ∇*ϕ*, in Eq. (15) can also be removed since the model utilizes the statistical information of regions, which has a larger capture range and capacity of anti-edge leakage. Finally, the level set formulation of the proposed model, Eq. (15), can be reduced as,
23

The main procedures of the proposed algorithm are summarized as follows:

Initialize the level set function ∅ as:
24

where *ρ* > 0 is a constant, Ω_0_ is a subset in the image domain Ω and ∂Ω_0_ is the boundary of Ω_0_.

**for** Check whether the evolution of the level set function has converged

Compute *C*_1_, *C*_2_, *f*_1_(*x*) and *f*_2_(*x*) using (4), (5), (9) and (10), respectively.

Evolve the level set function according to Eq. (23).

Let *ϕ* = 1 if *ϕ* > 0; otherwise, *ϕ* = - 1.

Regularize the level set function with a Gaussian filter, i.e. *ϕ* = *ϕ* ∗ *K*_*σ*_

**end**

## Results and discussion

The performance of our method is extensively evaluated in synthetic and real images. Particularly, We first describe the two-phase segmentation which compare the different models, and then show the application of multi-phase segmentation. In addition, the proposed method is implemented in Matlab R2009a on a 2.20GHz PC. Through the whole implementation, the parameters are described in the Table 1.

**Table 1:** **Description of the parameters used in the study**

Parameters	Functional description
*ρ*	To initialize the level set function. *ρ* > 0 is a constant.
*σ*	Scale parameter in Gaussian kernel (GCV: *σ* = 1; LBF: *σ* = 3; our model: *σ* = 5).
*λ*_1_	Inner weight of contour *C* (LBF: *λ*_1_ = 1).
*λ*_*2*_	Outer weight of contour *C* (LBF: *λ*_2_ = 1).
ϵ	The parameter of *H*_*3*_ and *δ*_*3*_ (LBF: *ϵ* = 1; our model: *ϵ* = 0.3).
*∆t*	Time step (GCV: *∆t* = 1; LBF: *∆t* = 0.1; our model: *∆t* = 1).
*β*	The weight of ω (our model: *β* = 1).
π	π = 3.14 is a constant.
*α*	Balloon force (GCV and our model: *α* determined according to images).

**Figure 4:**
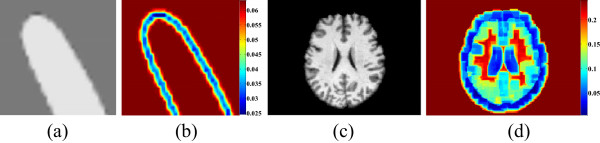
**Masks of ω value. (a)** Synthetic image; **(b)** ω value of **(a)**; **(c)** MR image; **(d)** ω value of **(c)**. In mask images, red to blue decreases gradually.

Besides these parameters, the parameter ω is a variable, which is calculated by using the local image contrast. When the intensity inhomogeneity is severe, the accuracy of segmentation relies on the local SPF. In such case, we choose small ω as the weight of the global SPF. While in the smooth regions, a bigger ω is chosen as the weight of global SPF so that the contour is attracted to the object boundary quickly. Figure 4(b) is the mask of ω value of a synthetic image and Figure 4(d) is the mask of ω value of a MR image. It can be seen from the two images that ω is bigger in the smooth regions and smaller in the regions close to boundary of objects. Therefore, the ω can adaptively adjust the global SPF term in all regions.

### Two-phase segmentation

Our method is first compared with the GCV and LBF model in the synthetic hand and the real blood images whose appearance show severe intensity inhomogeneity. And then our method is also compared in the real MR images to evaluate the performance of our method.

**Figure 5:**
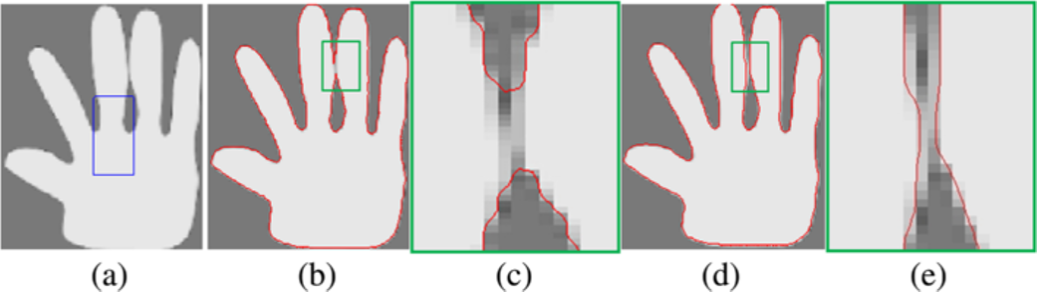
**Segmenting a hand phantom using the GCV and the proposed model.** Illustration of the performance of segmenting a hand phantom (downloaded from [13]) using the GCV and the proposed model: **(a)** initial contour, **(b)** segmentation result by the GCV model *α* = 20 **(c)** zoomed view of the narrow, green rectangle in (b), **(d)** segmentation result by our method, and **(e)** zoomed view of the narrow, green rectangle in **(d)**. The parameter *α* = 20.

Figure 5 shows that our method can achieve sub-pixel segmentation accuracy. Here, the blue rectangle in Figure 5(a) indicates the initial contour for all methods, and the red contours in (Figure 5(b) and (d)) denote the convergence results by GCV and our model, respectively. The regions surrounded by the green rectangle in (Figure 5(b) and (d)) are zoomed in (Figure 5(c) and (e)), respectively. We can see that our model can deal with intensity inhomogeneity region and the final contour covers the true hand shape, while GCV method make two middle fingers stick together, which is not desired. Here, the parameter α is set to 20.

**Figure 6:**
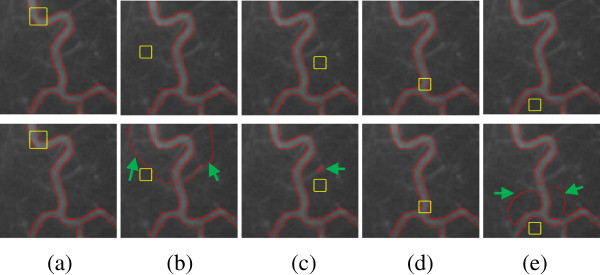
**Segmentation results on the real blood vessel image.** Yellow rectangle is the initial contours: Row 1 are the results by our model; Row 2 are the results by the LBF model; **(a)-(e)** are segmentation results in different initial contours. The parameter *α* = 10.

Figure 6 compares the performance of the LBF and our model in segmenting a real blood vessel image. The vessel image is of intensity inhomogeneity. The second row shows the results of the LBF model. We can see that the LBF can segment intensity inhomogeneity image on the condition that the initial contour, denoted as yellow rectangle, lies in the appropriate location such as (Figure 6(a) and (c)). While giving bad initial contour, a bad segmentation result will appear such as the contours indicated by the green arrows in (Figure 6(b), (d) and (e)). On the contrary, the first row shows that our model is less sensitive to the initial contour, and can achieve satisfying segmentation results. Despite the great difference of these initial contours, the corresponding results are almost similar to accurately capture the object boundaries. Furthermore, the iterations and CPU time of segmenting the image with size of 110 × 111 pixels in Figure 6 are listed in Table 2 for LBF model and our method. It can be observed that our method is much faster than LBF model. Accordingly, the proposed method is more efficient. Here, the parameter α is set to 10.

**Table 2:** **Iterations and CPU time (in seconds) needed by our model and LBF model when segmenting the image with the size of 110 × 111 in Figure** 6 **, respectively**

Method	Figure 6 (a)		Figure 6 (b)		Figure 6 (c)		Figure 6 (d)		Figure 6 (e)	
	Iterations	Time(s)	Iterations	Time(s)	Iterations	Time(s)	Iterations	Time(s)	Iterations	Time(s)
**Our model**	80	5.36	80	5.17	80	5.23	80	5.26	80	6.18
**LBF model**	200	12.01	200	13.65	120	8.24	120	8.20	200	14.05

**Figure 7:**
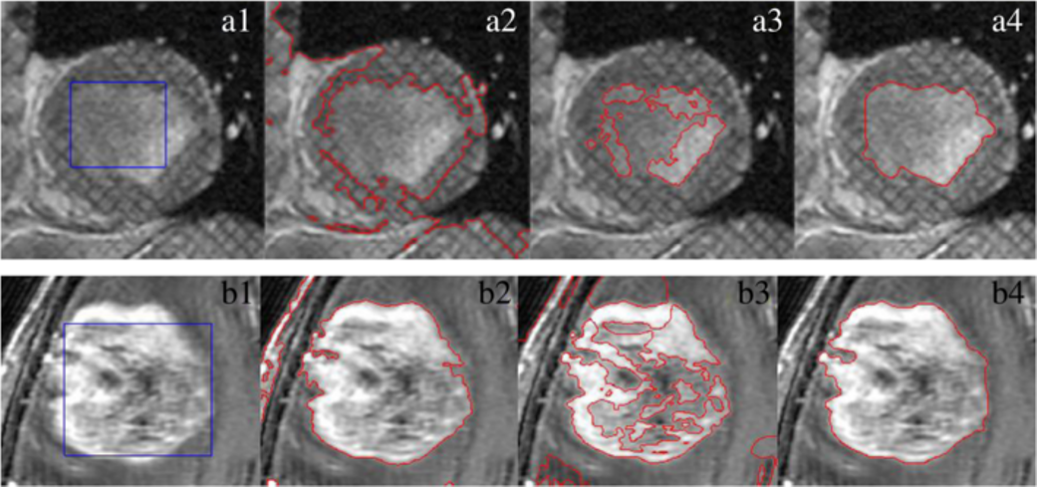
**Comparison of our method with GCV and LBF model in the segmentation of the two MR images in (a1) and (b1), respectively. (a1)**,**(b1)** blue rectangles are drawn as initial contours, **(a2)**, **(b2)** segmentation results from GCV, The parameter *α* = 10. **(a3)**, **(b3)** segmentation results from LBF model, and **(a4)**, **(b4)** segmentation results from our method, respectively. The parameter *α* = 10.

Two MR images with the intensity inhomogeneity shown in (Figure 7(a1) and (b1)) are used to evaluate the proposed model, GCV and LBF model, respectively. Here, the blue rectangles are drawn as the initial contour in (Figure 7(a1) and (b1)). (Figure 7(a2) and (b2)) indicate that the GCV model fails to segment the two images since it is based on only the global intensity information. (Figure 7(a3) and (b3)) show that LBF model is also failed in segmentation of the two images since it uses only the mean of local intensity. By contrast, the proposed method incorporating global and local intensity information successfully extracts object boundaries of the two MR images, as shown in (Figure 7(a4) and (b4)).

### Multi-phase segmentation of brain MR images

The segmentation of the brain MR images into WM, GM, and CSF is an important task in medical image analysis. A major difficulty in segmentation of MR images is the intensity inhomogeneity due to the noise. In this subsection, we will show an application of our multi-phase model to segment brain MR images. We also compare our method with the method of Li et al. [14] on 18 T1-w images obtained from the Internet Brain Segmentation Repository (IBSR) [19].

**Figure 8:**
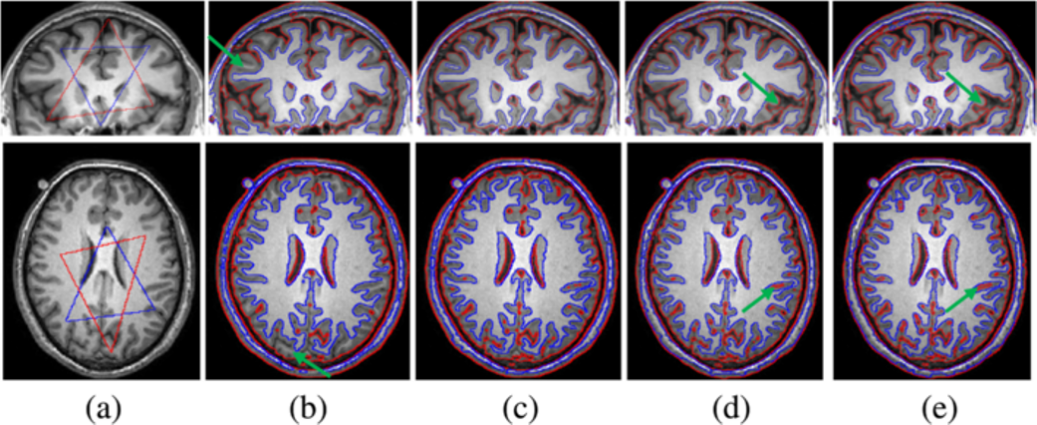
**Application of our method in segmenting the 3T MR images. (a)** original images and initial blue contours, **(b) (c) (d) (e)** segmentation results of ϵ is set to 0.1, 0.3, 0.5, 0.8, respectively. The parameter *α* = 30.

Frist, we apply our multiphase model to segment two 2D MR images. The initial blue and red contours are displayed in Figure 8(a). The segmentation results of different ϵ values are shown in Figure 8(b)-(e), respectively. The number of iterations is 50. It can be observed that WM, GM, and CSF are well segmented by our method. From Figure 8(c) we can see that the tissue and the background of the image can be well-separated, which make our method have a strong discriminative capability for the tissue and the background. In contrast, for the case (b), (d) and (e), the results (Figure 8(b) (d) and (e)) are not consistent with the anatomy of brain in some areas. Such as those pointed by the green arrows. For example, WM is mislabeled as GM in Figure 8(b) and GM is mislabeled as CSF in (Figure 8(d) and (e)). Here, ϵ is set 0.3 in our model and the parameter α is set to 50.

**Figure 9:**
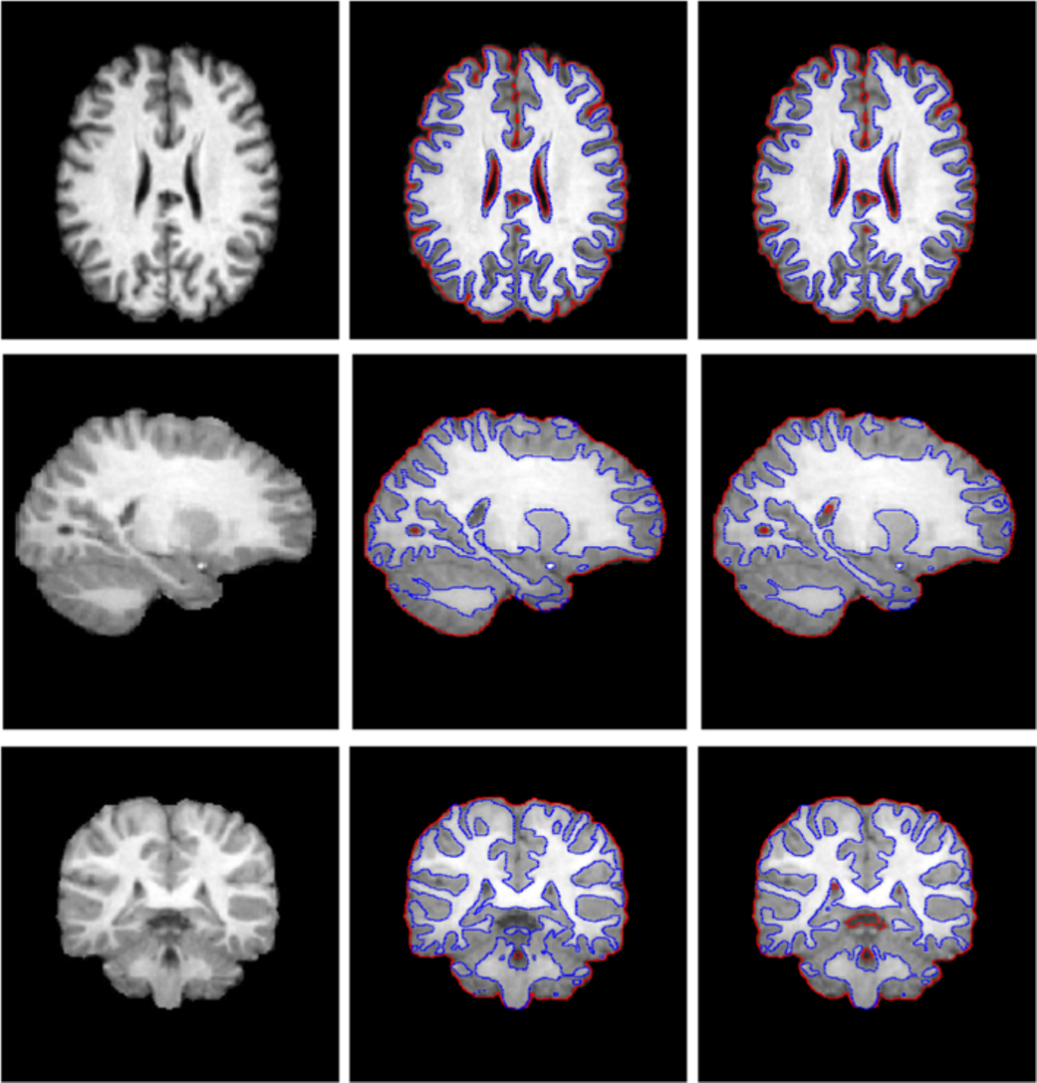
**Comparison our method with Li’s method by using the data from IBSR.** Colum 1: original images (row 1: Axial plane, row 2: sagittal plane, row 3: coronal plane); Colum 2: results of Li’s method; Colum 3: results of our method. The parameter *α* = 100.

**Figure 10:**
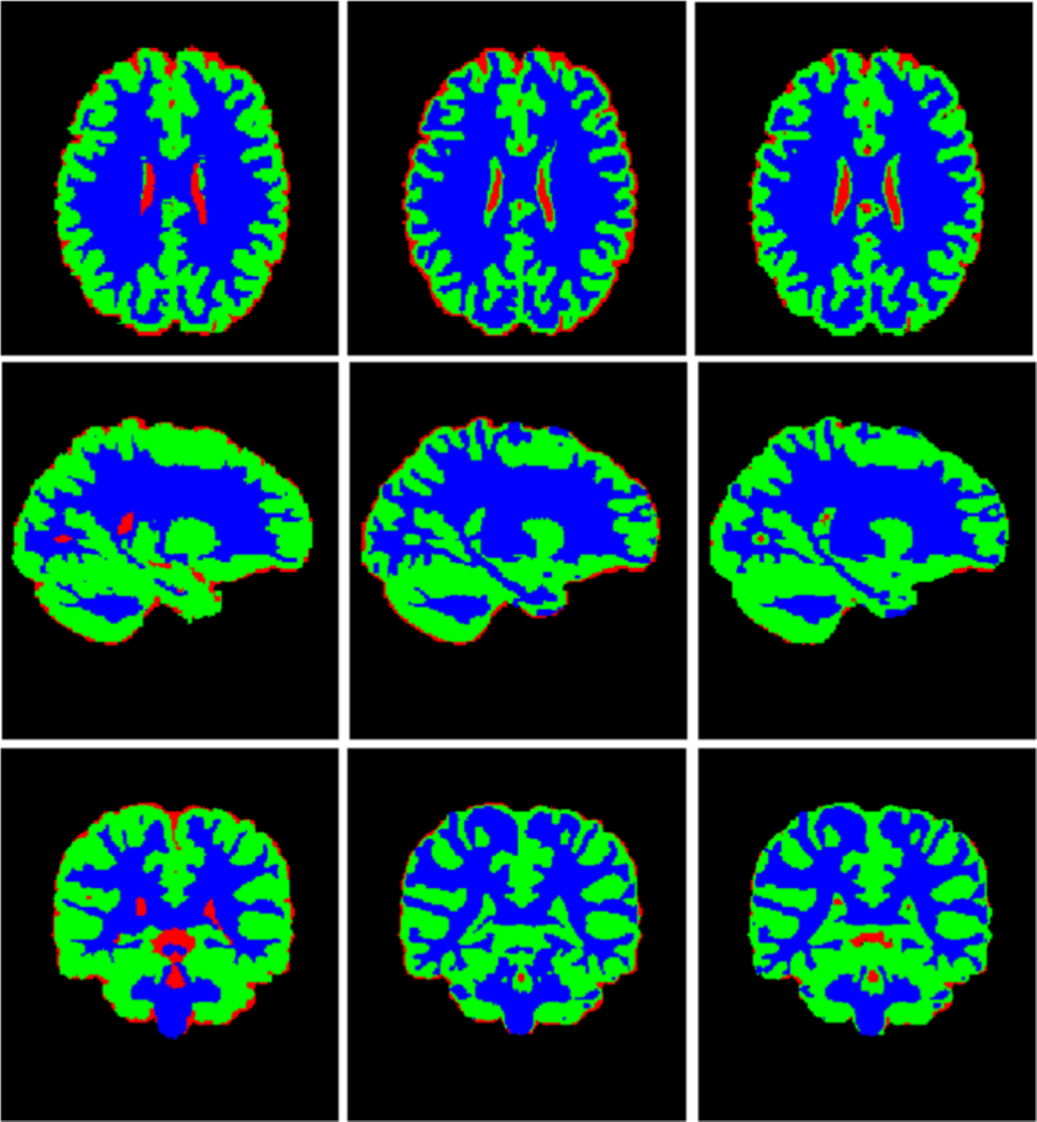
**Tissue Segmentation masks of Figure** 8 **.** Colum 1: ground truth; Colum 2: Li’s method; Colum 3: our method.

Second, Figure 9 and Figure 10 shows the comparison between the proposed method and Li’s method. Figure 9 displays the segmentation results by estimating . The first Colum in Figure 10 shows the ground truth segmentation from IBSR [19]. It can be observed that the results by our model and Li’s method look similar by visual comparison. We use the Dice Similarity Coefficient (*DSC*) [20] as an index to measure the segmentation accuracy of WM, GM and CSF, which is defined as

**Figure 11:**
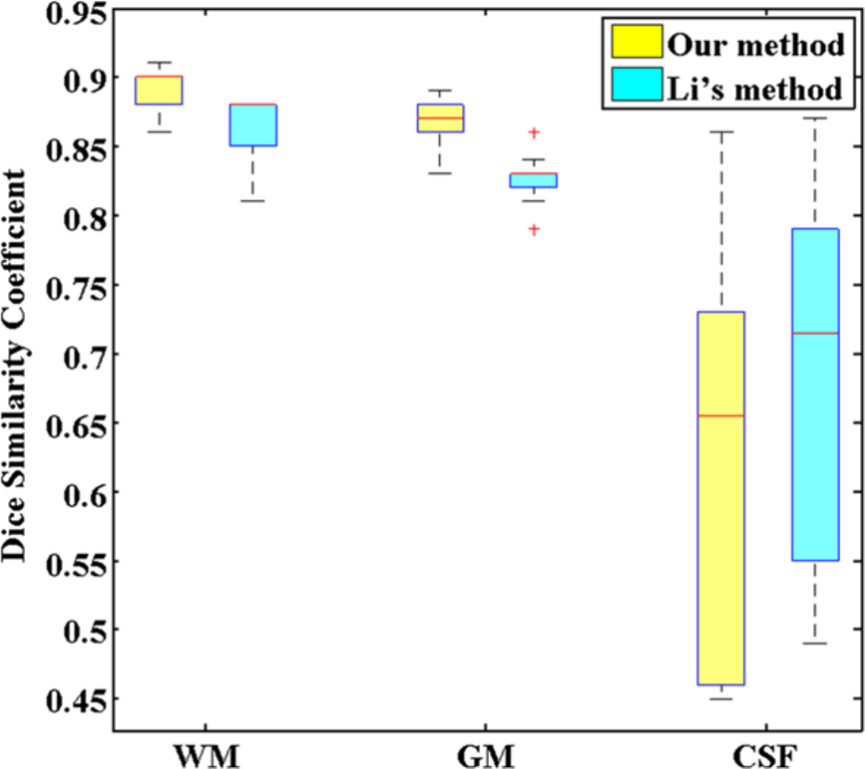
**Box plot of the** ***DSC*** **values of WM, GM and CSF by our method and Li’s method, respectively.**

25

where *S*_*1*_ and *S*_*2*_ represent the obtained segmentation and ground truth, respectively, and N(·) indicates the number of voxels in the enclosed set. The closer the *DSC* value to 1, the better the segmentation. Table 3 and Figure 11 shows the *DSC* values of WM, GM and CSF by the two methods, respectively. It can be seen that our method can achieve more accurate results by comparing with Li’s method in WM and GM. The third column in Figure 10 shows that our method have a bad performance in segment CSF on the edge of images. So the *DSC* values of CSF by our method is lower than Li’s method. The p-value of statistical significance of the improvement of our method over Li’s method are less than 0.0005 when using a paired student t-test, which shows that our method significantly outperforms Li’s method with higher accuracy in terms of *DSC* results. Similarly, compared with Sergi et al. research [21], our method also has relatively high accuracy. Here, the parameter *α* is set to 100.

**Table 3:** **The average** ***DSC*** **values of WM, GM and CSF by the two methods, respectively**

Tissue	WM	GM	CSF
**Li’s method**	0.86 ± 0.03	0.83 ± 0.02	0.68 ± 0.14
**Our method**	0.89 ± 0.01	0.87 ± 0.02	0.63 ± 0.14

(Data layout: mean ± std.).

## Conclusion

This paper presented a novel segmentation model by incorporating the local and global information into the original GAC model. Particularly, a new local SPF function is used to capture the local intensity information, so the novel model is especially fit for the segmentation of the inhomogeneous images. The weight balancing the global term is adaptively adjusted according to the statistics of the local intensity information. In a word, the proposed model can not only allow flexible initialization but also estimate intensity inhomogeneity. Moreover, the proposed method has better efficiency since it reduces the expensive re-initialization of the traditional level set method. In the future, the proposed method will be evaluated in the more extensive experiments.
